# Comparison of the Commercial Color LCD and the Medical Monochrome LCD Using Randomized Object Test Patterns

**DOI:** 10.1371/journal.pone.0037769

**Published:** 2012-05-31

**Authors:** Jay Wu, Tung H. Wu, Rou P. Han, Shu J. Chang, Cheng T. Shih, Jing Y. Sun, Shih M. Hsu

**Affiliations:** 1 Department of Biomedical Imaging and Radiological Sciences, College of Health Care, China Medical University, Taichung, Taiwan, Republic of China; 2 Department of Biomedical Imaging and Radiological Sciences, National Yang-Ming University, Taipei City, Taiwan, Republic of China; 3 Department of Management Information Systems, Central Taiwan University of Science and Technology, Taichung, Taiwan, Republic of China; 4 Health Physics Division, Institute of Nuclear Energy Research, Longtan, Taiwan, Republic of China; 5 Department of Biomedical Engineering and Environmental Sciences, National Tsing-Hua University, Hsinchu, Taiwan, Republic of China; Glasgow University, United Kingdom

## Abstract

Workstations and electronic display devices in a picture archiving and communication system (PACS) provide a convenient and efficient platform for medical diagnosis. The performance of display devices has to be verified to ensure that image quality is not degraded. In this study, we designed a set of randomized object test patterns (ROTPs) consisting of randomly located spheres with various image characteristics to evaluate the performance of a 2.5 mega-pixel (MP) commercial color LCD and a 3 MP diagnostic monochrome LCD in several aspects, including the contrast, resolution, point spread effect, and noise. The ROTPs were then merged into 120 abdominal CT images. Five radiologists were invited to review the CT images, and receiver operating characteristic (ROC) analysis was carried out using a five-point rating scale. In the high background patterns of ROTPs, the sensitivity performance was comparable between both monitors in terms of contrast and resolution, whereas, in the low background patterns, the performance of the commercial color LCD was significantly poorer than that of the diagnostic monochrome LCD in all aspects. The average area under the ROC curve (AUC) for reviewing abdominal CT images was 0.717±0.0200 and 0.740±0.0195 for the color monitor and the diagnostic monitor, respectively. The observation time (OT) was 145±27.6 min and 127±19.3 min, respectively. No significant differences appeared in AUC (*p* = 0.265) and OT (*p* = 0.07). The overall results indicate that ROTPs can be implemented as a quality control tool to evaluate the intrinsic characteristics of display devices. Although there is still a gap in technology between different types of LCDs, commercial color LCDs could replace diagnostic monochrome LCDs as a platform for reviewing abdominal CT images after monitor calibration.

## Introduction

Over the last decade, picture archiving and communication systems (PACSs) have gradually replaced traditional methods of managing and displaying medical images. A workstation with electronic display devices is an essential component in the PACS. It not only servers as the main medium for accessing medical images, but also provides radiologists with a convenient platform for diagnosis [1]. The performance of display devices in consideration of visual characteristics should be verified to ensure that image quality is not jeopardized.

Previous reports suggest that healthcare institutions should assure the image quality associated with display devices to avoid consequent medical-legal problems [2]. Digital Imaging and Communications in Medicine Part 14 (DICOM PS 3.14) recommends the use of the grayscale standard display function (GSDF) [3] as an output reference for calibrating the luminance of a display system. This enables inter- and intra-institutional comparisons practicable on the basis of the consistent presentation of radiographic images. The GSDF has also been applied to evaluate the angular dependency of luminance in liquid crystal displays (LCDs) [4], [5], since a limited viewing angle is a major shortcoming of the LCD technology.

Researchers have developed various image test patterns to evaluate the physical properties of display devices. The American Association of Physicists in Medicine Task Group 18 (AAPM TG-18) [6] has specified a series of test patterns and standard procedures for assessing the geometric distortion, display reflection, luminance response, display resolution, and other characteristics of display devices. The Society of Motion Picture and Television Engineers (SMPTE) test patterns [7] represents another popular branch broadly applying to image processing [8], [9] and display performance evaluating [10], [11]. Additionally, a new grayscale test pattern (NGTP) was developed to adjust the gradient of gray levels in thoracic computed tomography (CT) images [12]. The pattern has also been applied to the psychophysical evaluation of display functions for LCD monitors [13], [14].

Considering product costs and recent advances of commercial color monitors, many studies have evaluated the opportunity to replace the medical monochrome LCDs with the commercial color LCDs for specific disease diagnosis using observer studies [15]–[18]. The results commonly reveal that using commercial color LCDs does not compromise the diagnostic accuracy. However, distinguishing the differences between these two types of monitors remains challenging because it is difficult to gather images with subtle lesions, even in a medical center. The purpose of this study is two-fold: first, to create a series of randomized object test patterns (ROTPs) to differentiate the physical characteristics of monochrome and color LCDs, and second, to evaluate the possibility of replacing medical monochrome LCDs with commercial color LCDs as a diagnostic platform for reviewing abdominal CT images using the ROTPs.

## Materials and Methods

### Display devices

Two types of LCD monitors were attached separately to a Windows-based PACS workstation. One was a 3-mega-pixel (MP) diagnostic monochrome LCD (Barco E-3620, Kortrijk, Belgium), having a viewable size of 21 inches and a resolution of 1,536×2,048 pixels. This monochrome LCD belonged to the primary display system and was regarded as the standard for reviewing medical images. The other one was a commercial color LCD monitor (EZIO FlexScan S2431W, Ishikawa, Japan) with a 1,920×1,200 resolution operated in the portrait orientation. Table 1 lists their detailed physical specifications and panel types. Images were downloaded from an image Web server (Siemens MagicWeb VA40A, Malvern, Pa) and displayed on both monitors.

**Table 1:** Physical specifications of the diagnostic monochrome LCD and the commercial color LCD.

	Monochrome LCD	Color LCD
Size	20.8 inch	24.1 inch
Display mode	portrait	landscape/portrait*
Resolution	1,536×2,048	1,200×1,920
Pixel size	0.207×0.207 mm^2^	0.270×0.270 mm^2^
Color depth	10 bit grayscale	24 bit color
Luminance	700 cd/m^2^	360 cd/m^2^
Contrast	900∶1	1,000∶1
Panel type	PVA	DIPS

* Displayed in the portrait shaped mode throughout this study.

The workstation and the display devices were turned on for at least 30 minutes before each reviewing session. An illuminance meter (RSR LX-101, Avenel, NJ) was used to ensure the ambient lighting level was lower than 10 lm/m^2^ (lux) to minimize artifacts and loss of image quality due to faceplate reflection. Following the AAPM TG-18 procedures [6], the luminance response of each monitor was calibrated to the DICOM GSDF using a telescopic photometer (TOPCON BM-7A, Tokyo, Japan) and TG-18 LN test patterns. The maximum difference between the measured and GSDF contrast responses was within 20% for the commercial color LCD and 10% for the medical monochrome LCD, respectively, as suggested by TG-18.

### Randomized object test patterns

**Figure 1:**
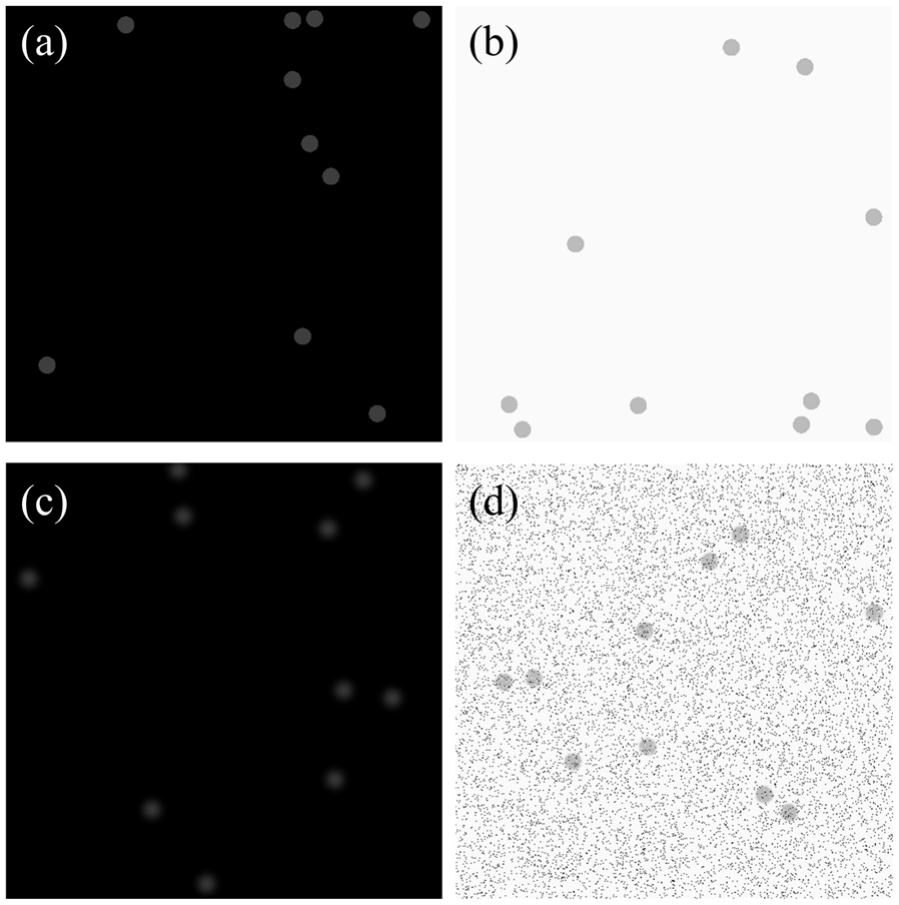
Examples of various ROTPs. The image patterns showed in the display devices for (a) the contrast test in the low background condition, (b) the resolution test in the high background condition, (c) the point spread test in the low background with *σ* of 5, and (d) the noise test with noise density of 0.1 in the high background.

A series of randomized object test patterns (ROTPs), each with 512×512×12 bits, was dynamically produced by MATLAB (Mathworks, Natick, Massachusetts). Spheres with various numbers, sizes, and gray levels were randomly added to the patterns to evaluate different characteristics of the LCD displays. The test items, consisting of the image contrast, resolution, point spread effect, and noise, were analyzed in the low and high background conditions, for which the gray intensity was 0 and 4000, respectively (Fig. 1). Once the ROTPs were created, they were uploaded to the PACS image server as DICOM image objects and accessed from the intranet without compression. No additional image processing functions and lookup tables (LUT) were applied to the ROTP patterns after they were downloaded. A homemade graphical user interface was installed in the display workstation to evaluate the visibility of spheres through mouse clicking.

### Sensitivity analysis

Each test pattern randomly included five to twenty spheres, and several test patterns with a total of 100 spheres were sequentially displayed on the monitor. Four consequences, including true positive (TP), false positive (FP), true negative (TN), and false negative (FN), were identified as a result of reviewers' clicking. The sensitivity was calculated by the following equation:
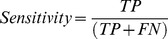
(1)In the contrast test, the radius of the spheres was 10 pixels and the grayscale was varied successively. In the resolution test, the grayscale of the spheres was set at 300 and 3700 for the low and high background conditions, respectively. The diameter of the spheres varied from 1 to 12 pixels. In the point spread test, the spheres with 10-pixel radius were convoluted with a 5×5 Gaussian function with various standard deviations (*σ*) to simulate the blurring effect of the imaging system. Lastly, the noise test patterns possessed the properties of the point spread patterns but had a fixed *σ* = 5. Pepper noise and salt noise were added to the high and the low background conditions, respectively. The noise density was varied to simulate the effect of impulse disturbances. The sensitivity differences between the two LCD monitors were compared using the chi-square test.

### Simulation of abdominal CT images

**Figure 2:**
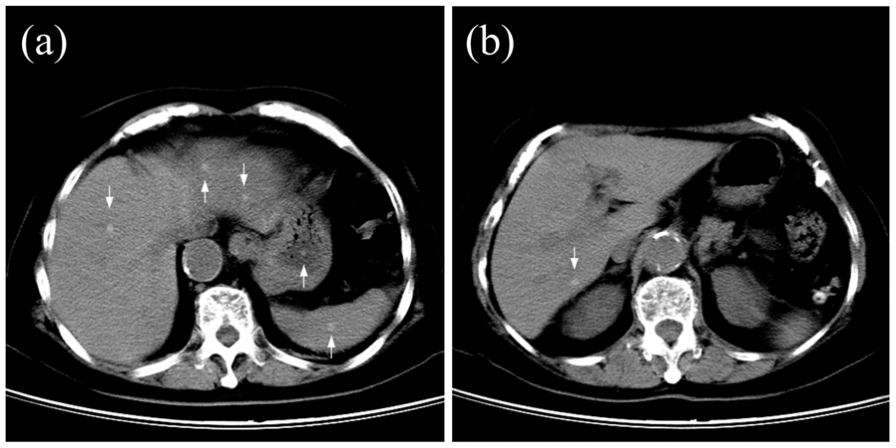
Abdominal CT images combined with the ROTPs. Several spheres were merged with the CT images: (a) 3 to 5 spheres as the positive group and (b) 0 to 2 spheres as the negative group.

A total of 120 normal abdominal CT images were selected from the PACS archive server. Among them, 60 images were merged with 3 to 5 spheres and classified as the positive group, while the rest were merged with 0 to 2 spheres and classified as the negative group (Fig. 2). All spheres had a diameter of three pixels, 50 Hounsfield Units (HU) higher than the background, and point spreading with *σ* = 6. Five radiologists with at least two years of experience were asked to review the images on the monochrome and color LCDs, respectively. They were allowed to adjust the window width/level arbitrarily without time restriction. The time interval between reviewing two monitors was longer than two weeks, and the images were randomly rearranged for each evaluation.

### ROC analysis

A five-point rating scale was used for scoring, where a score of one represented “definitely negative finding” and a score of five represented “definitely positive finding.” The reviewers were also asked to label the location of the spheres in the images. The ROC analysis was performed using the MedCalc statistical software (Version 11, Mariakerke, Belgium). The area under curve (AUC) was calculated to determine the diagnostic accuracy. The difference between the mean AUCs was calculated using the method proposed by DeLong et al [19] based on the 95% confidence interval. The observation time (OT) for each review section was recorded as well.

## Results

**Figure 3:**
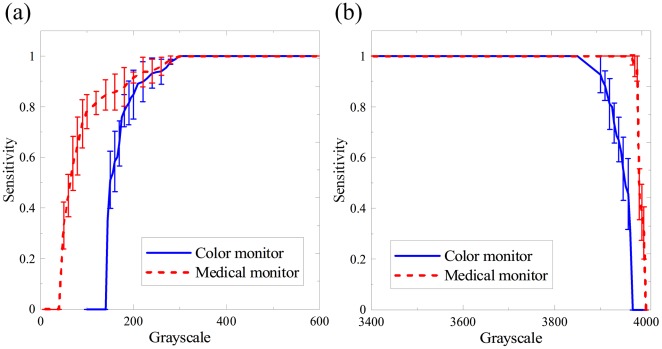
The average contrast-sensitivity curves for different LCDs. (a) Under the low background, the grayscale at the threshold of 80% sensitivity was 188 and 113 for the color and monochrome LCDs, respectively. (b) Under the high background, the sensitivities for both monitors reached 100% when the grayscale difference between the background and the sphere exceeded 150. In Figs. 3–6, error bars show standard error, n = [100].

Figure 3 shows the average contrast-sensitivity curves where the error bar represents the scattering within one standard error around mean. For the low background patterns, the grayscale corresponding to 80% sensitivity was 113 and 188 for the monochrome and color LCDs, respectively, indicating that the former had better contrast discrimination ability than the latter. As for the high background patterns, the two curves decreased rapidly as the contrast between the sphere and background decreased. The sensitivities of the two monitors were both 100% when the grayscale difference exceeded 150. This means that both monitors have comparable performance under the high background condition, and it is easier to identify subtle changes in gray level under the high background condition than the low background condition.

**Figure 4 pone-0037769-g004:**
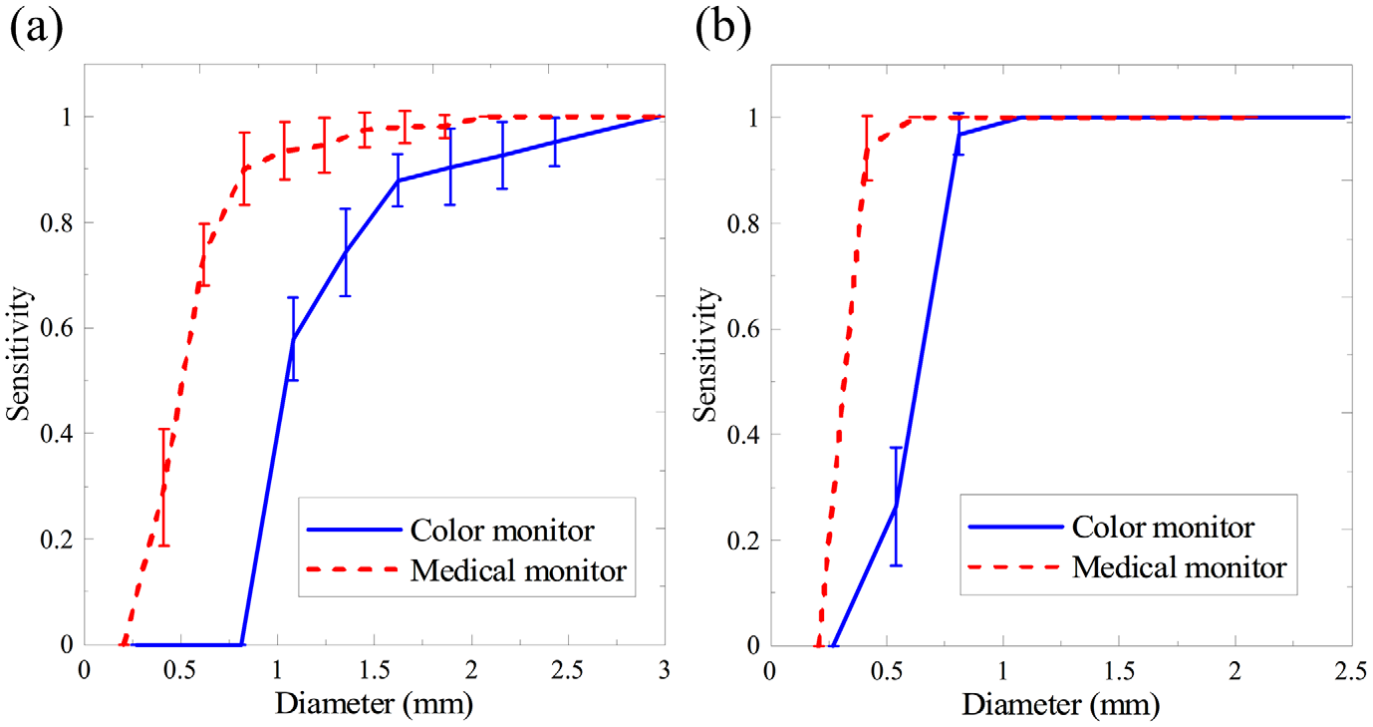
The average resolution-sensitivity curves for different LCDs. (a) Under the low background, the diameter corresponding to 80% sensitivity was 1.485 mm and 0.725 mm for the color and monochrome LCDs, respectively. (b) Under the high background, when the diameter exceeded 1 mm, no errors can be found with both monitors.

Figure 4 depicts the relationship between the average sensitivity and the actual object size displayed on the screen which is the product of the pixel number and pixel size. The commercial color LCD required 1.485 mm for the 80% sensitivity, while the medical monochrome LCD required 0.725 mm in diameter under the low background level. This result verifies that the medical LCD has better spatial resolution than the commercial LCD. Under the high background condition, the diameter difference between monitors was decreased markedly to 0.357 mm at the 80% sensitivity level. When the diameter exceeded 1 mm, both monitors had no observation errors.

**Figure 5 pone-0037769-g005:**
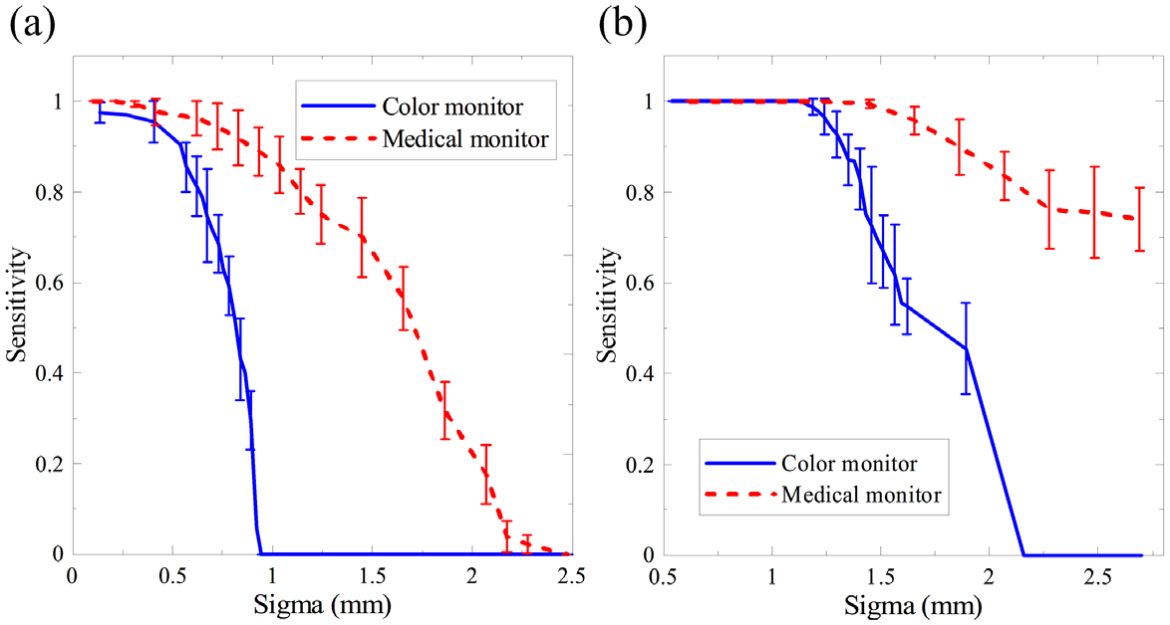
The average sensitivity curves for the point spread test under (a) the low background and (b) the high background conditions. As the *σ* increased, the curve slope of the commercial color LCD dropped more dramatically than the medical monochrome LCD.

Figure 5 illustrates the average sensitivity dependency on the standard deviation of the point spread function. In both conditions, the curves of the color LCD dropped more rapidly than those of the medical monochrome LCD as *σ* increased. Both monitors exhibited higher tolerance of *σ* for the high background condition than for the low background condition. The *σ* at the threshold of 80% sensitivity significantly increased by 0.756 mm for the color LCD and 0.994 mm for the monochrome LCD, respectively. This indicates that the effect of image blurring can be suppressed in the high background situation.

**Figure 6:**
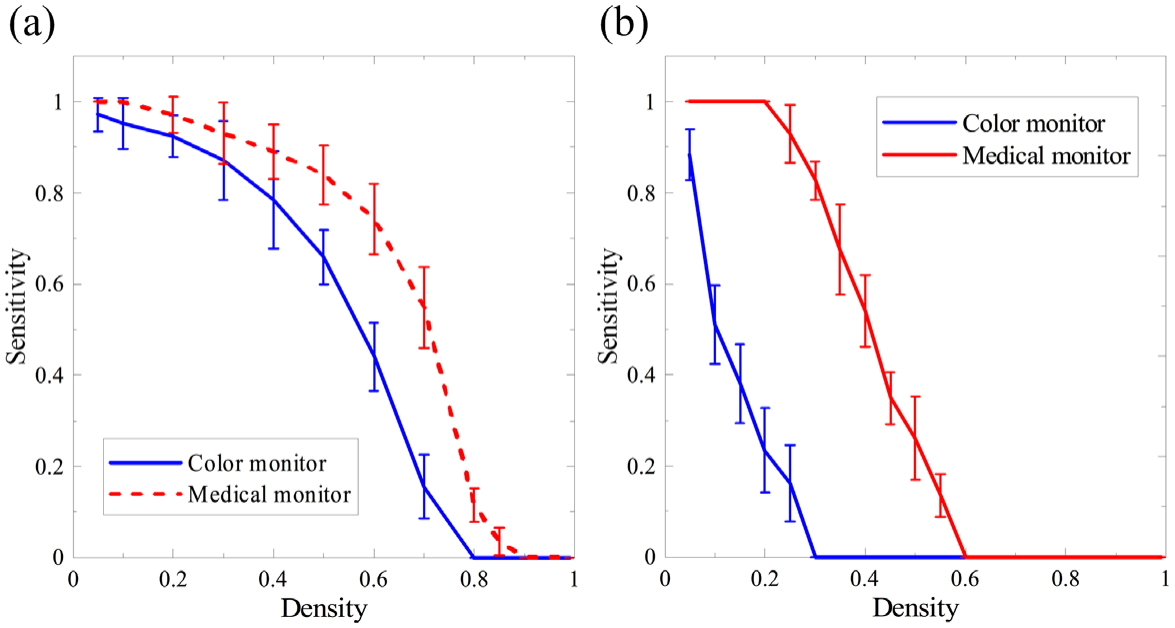
The noise-sensitivity relationship for different LCDs. (a) Salt noise was added to the low background pattern, and (b) pepper noise was added in the high background pattern.

Figure 6 shows the relationship between the average sensitivity and the noise density. When salt noise was intentionally added to the low background pattern, the tolerable density of noise distribution was 0.55 for the monochrome LCD at the sensitivity level of 80%, and 0.38 for the color LCD. When pepper noise was added to the high background pattern, the monochrome LCD again surpassed the color LCD in performance. However, the tolerable noise density decreased to 0.31 and 0.05 respectively, implying that pepper noise affects image quality more heavily than salt noise. In other words, even a small amount of pepper noise can jeopardize the image quality in the high background condition when using the color LCD.

**Figure 7:**
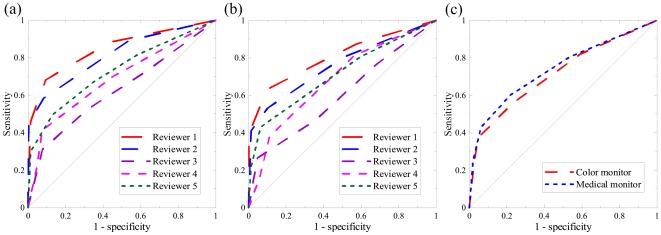
The ROC curves of five individual reviewers for (a) the medical monochrome LCD and (b) the commercial color LCD. (c) The average AUC was 0.717 and 0.740 for the color and monochrome LCDs, respectively. No statistical significance can be found (*p* = 0.265).

The ROTP test results provide a set of parametric combinations that shows significant differences between the average sensitivity curves of the two monitors (Table 2). This parametric setting was used to randomly add spheres to abdominal CT images. Figure 7 illustrates the ROC curves of five individual reviewers and average ROC curves for each LCD, while Table 3 lists the AUC and the OT for each reviewer. Though the average AUC of the color LCD was 3.1% lower than that of the monochrome LCD, the difference did not achieve statistical significance (*p* = 0.265). In addition, the average OT for the color LCD was approximately 14% higher than that for the monochrome LCD. Based on the paired samples *t*-test, this difference was not statistically significant (*p* = 0.07).

**Table 2:** Parametric combinations showing significant differences between the monochrome and color LCDs using Chi-square analysis.

	Low background	High background
Contrast (grayscale)	50–100	3960–3980
Resolution (pixel)	2–3	3
Point spread (pixel)	4–6	7–8
Noise (%)	0.7	0.2–0.5

**Table 3:** AUC and OT for each individual reviewer.

	Monochrome LCD		Color LCD	
Reviewer	AUC	OT	AUC	OT
1	0.849	121	0.811	160
2	0.817	109	0.765	118
3	0.631	113	0.604	112
4	0.687	155	0.682	166
5	0.729	139	0.714	169
Average	0.740±0.0195	127±19.26	0.717±0.0200*	145±27.66**

AUC stands for the area under curve; OT stands for the observation time in min.

* No significant difference (*p* = 0.265).

** No significant difference (*p* = 0.07).

## Discussion

The ROTP results reveal that both LCDs achieved comparable performance in the high background condition of contrast and resolution. This may be because although within the criteria of TG-18, the commercial LCD has a slightly higher contrast response than the expected GSDF response at the high just noticeable difference (JND) index [3]. In this case, the luminance contrast in the low background situation could be compromised. Therefore, if a commercial color LCD is used for image diagnosis, applying complement transformation functions may be an effective way to improve the efficacy of diagnostic discrimination.

As for the other ROTP tests, the sensitivity performance of the color LCD was poorer than that of the monochrome LCD. This is primarily due to differences in the intrinsic physical specifications, including the poorer native spatial resolution, grayscale depth, and maximum luminance. These unfavorable conditions can be conquered by adjusting the window width/level, which is also why the average OT for the color LCD is longer than that for the medical LCD. This result agrees with the findings of Wang et al [20]. However, due to the large inter-observer variability in OT, the difference between the two monitors is not statistically significance.

Luminance is an important aspect of display performance. The difference between the maximum luminance *L*_max_ and the minimum luminance *L*_min_ determines the dynamic range of JND. Therefore, the lower the *L*_max_, the poorer the grayscale resolution [21]. According to the manufacturer, the *L*_max_ of the color LCD used in this study is 360 cd/m^2^. Although this exceeds the criterion of 171 cd/m^2^ suggested by the American College of Radiology (ACR) [22], it does not meet the optimized contrast guideline of 450 cd/m^2^. A larger *L*_min_ and ambient luminance *L*_amb_ also decrease the dynamic range of contrast and compromise the luminance ratio. Since the *L*_max_ and *L*_min_ are fixed at the given brightness control of the display device, decreasing ambient lightening is necessary to improve the grayscale resolution of the commercial color LCD.

The purpose of introducing the ROTPs was to distinguish the differences between monochrome and color LCDs. However, the discrepancy cannot be separated statistically using ROC analysis when the ROTPs were merged with abdominal CT images. In other words, although the physical performance of the color LCD is slightly less due to its inferior specifications, it does not decrease diagnostic performance in reviewing abdominal CT images. However, the color LCD has the advantages of low cost, high interchangeability, and good integration ability with other pseudocolor images. Therefore, if the characteristics of color LCDs can be validated by the TG-18 protocol or ROTPs, color LCDs have the potential to substitute medical LCDs in PACSs for reviewing abdominal CT images.

As display technology continues to advance, the performance of commercial LCDs is becoming better and better. Spatial resolution has reached W-QUXGA (3840×2400) with a color depth of 32 bits per pixel and a maximum luminance of more than 500 cd/m^2^. Therefore, commercial color LCDs may also be used for reviewing other medical images which have higher resolution, such as mammography and digital radiography. In the future, the ROTPs can be applied as a quality control tool to establish the acceptance criteria for primary and secondary display systems. The ROTPs can also be used to evaluate the possibility of replacing medical monochrome LCDs with commercial color LCDs for other types of medical images and diseases.

### Conclusion

The proposed ROTPs can easily be integrated into the quality control procedure of medical display devices as subjective indices to reflect visual perception. The evaluation process does not need any special instruments, such as the telescopic photometer and illuminance meter. Results show that even though the intrinsic characteristics of the color LCD are poorer than those of the diagnostic monochome LCD in terms of image contrast, resolution, noise and point spread effect, the difference in diagnostic accuracy of both devices is not statistically significant using ROC analysis. This implies that with appropriate monitor calibration, commercial color LCDs have the potential to replace medical-grade diagnostic monochrome LCDs as the alternative platform for interpreting abdominal CT images.
